# Atypical Neurological Manifestations of COVID-19

**DOI:** 10.7759/cureus.8518

**Published:** 2020-06-08

**Authors:** Ishita Gupta, Mithun K Reddy, Mir Mehdi Hussain, Pooja M Murthy, Chris A Robert

**Affiliations:** 1 Medicine, Dr. Rajendra Prasad Government Medical College, Kangra, IND; 2 Medicine, Vydehi Institute of Medical Sciences and Research Centre, Bangalore, IND; 3 Obstetrics & Gynecology, Sunrise Hospital, Pune, IND

**Keywords:** coronavirus, covid-19, neurological signs and symptoms, cns manifestations, sars-cov-2

## Abstract

The novel coronavirus (SARS-CoV-2), belonging to a group of RNA-enveloped viruses and believed to be transmitted by aerosol route, is a worldwide pandemic. Many studies have described typical clinical manifestations such as fever, cough, fatigue, diarrhea, and nasal congestion. However, to our knowledge, there are minimal studies on the neurological manifestations in SARS-CoV-2 positive patients. Our review aims to identify the various neurological manifestations in SARS-CoV-2 positive patients, which could be an added advantage in the early diagnosis and prevention of further complications of the nervous system.

## Introduction and background

Over the past few months, we have witnessed the unfolding of the COVID-19 pandemic. There are over 5 million confirmed cases worldwide; we must remember that these are people and not just numbers. There is a wide array of presentations of COVID-19. It is necessary to understand the timing and relation of neurological manifestations associated with SARS-CoV-2 [1]. Expanding anecdotal evidence suggests an increase in cases of anosmia during the global pandemic, suggesting that olfactory dysfunction can be caused by COVID-19 [2]. It has been shown that the SARS-CoV-2 binds to the ACE-2 (angiotensin-converting enzyme-2) receptors and invades the cell; the presence of the ACE-2 receptor on the neurological tissue poses a potential risk of neurologic tissue damage in individuals with severe COVID-19 infections [3]. It has been observed that, along with typical respiratory complaints, clear neurological manifestations, such as anosmia, ageusia, ataxia, and convulsions, have been reported in patients with COVID-19 [4]. Researchers have confirmed the presence of SARS-CoV-2 in cerebrospinal fluid by genome sequencing, and attempts to isolate the virus from that fluid could determine the fate of the virus or the host in this potentially fatal course of the illness [5].

## Review

Methods

Search Method and Strategy

We conducted a systematic search during March and April of 2020 for research articles on the neurological manifestations of COVID-19. Three primary databases were used: PubMed, Google Scholar, and the WHO. The search strategy used the keywords coronavirus, COVID-19, neurological signs and symptoms, and CNS manifestations and was comprehensive with cross-checking reference lists from the articles retrieved. The MeSH keywords used were coronavirus, COVID-19, neurological signs and symptoms, and CNS manifestations and their combinations.

Data Screening and Eligibility

The final review articles fulfilled the following criteria:

1. Reported neurological findings in COVID-19 positive patients

2. Included patient data regardless of age, gender, or location

3. Published in English

4. Qualified as full text, peer-reviewed articles

Articles that did not contain patient data or studies pertaining to SARS-CoV-1 and MERS were excluded. In doing so, we had 20 articles (see Table 1) for the final review. Each paper was reviewed by two reviewers independently, and disagreements were discussed among all reviewers and resolved via a consensus.

**Table 1:** Various studies included in our review

Article title	Author	DOI	Journal
Neurological Complications of Coronavirus Disease (COVID-19): Encephalopathy	Filatov et al. [6]	10.7759/cureus.7352	Cureus
Olfactory and gustatory dysfunctions as a clinical presentation of mild-to-moderate forms of the coronavirus disease (COVID-19): a multicenter European study	Lechien et al. [7]	10.1007/s00405-020-05965-1	Springer
Isolated sudden onset anosmia in COVID-19 infection. A novel syndrome?	Gane et al. [8]	10.4193/Rhin20.114	Rhinology Journal
Neurological manifestations of hospitalized patients with COVID-19 in Wuhan, China: a retrospective case series study	Mao et al. [9]	10.2139/ssrn.3544840	JAMA Neurology
Self-reported olfactory and taste disorders in patients with SARS-CoV-2 infection: a cross-sectional study	Giacomelli et al. [10]	10.1093/cid/ciaa330	Clinical Infectious Disease
A first case of meningitis/encephalitis associated with SARS-coronavirus-2	Moriguchi et al. [11]	10.1016/j.ijid.2020.03.062	International Journal of Infectious Diseases
COVID-19-associated acute hemorrhagic necrotizing encephalopathy: CT and MRI features	Poyiadji et al. [12]	10.1148/radiol.2020201187	Radiology
Guillain-Barré syndrome associated with SARS-CoV-2 infection: causality or coincidence?	Zhao et al. [13]	10.1016/S1474-4422(20)30109-5	The Lancet
Sudden and complete olfactory loss function as a possible symptom of COVID-19	Eliezer et al. [14]	10.1001/jamaoto.2020.0832	Jama Network
Hearing loss and COVID-19: a note	Sriwijitalai and Wiwanitkit [15]	10.1016/j.amjoto.2020.102473	American Journal of Otolaryngology
Characteristics of ocular findings of patients with coronavirus disease 2019 (COVID-19) in Hubei Province, China	Wu et al. [16]	10.1001/jamaophthalmol.2020.1291	Jama Network
Alterations in smell or taste in mildly symptomatic outpatients with SARS-CoV-2 infection	Spinato et al. [17]	10.1001/jama.2020.6771	Jama Network
COVID-19 may induce Guillain-Barré syndrome	Camdessanche et al. [18]	10.1016/j.neurol.2020.04.003	Revue Neurologique
Guillain-Barré syndrome following COVID-19: new infection, old complication?	Padroni et al. [19]	10.1007/s00415-020-09849-6	Springer
Neurologic manifestations in an infant with COVID-19	Dugue et al. [20]	10.1212/WNL.0000000000009653	Neurology
Meningoencephalitis without respiratory failure in a young female patient with COVID-19 infection in Downtown Los Angeles, early April 2020	Duong et al. [21]	10.1016/j.bbi.2020.04.024	Brain, Behaviour and Immunity
Concomitant neurological symptoms observed in a patient diagnosed with coronavirus disease 2019	Yin et al. [22]	10.1002/jmv.25888	Journal of Medical Virology
Acute‐onset smell and taste disorders in the context of Covid‐19: a pilot multicenter PCR‐based case‐control study	Beltrán‐Corbellini [23]	10.1111/ene.14273	European Journal of Neurology
Guillain Barre syndrome associated with COVID-19 infection: a case report	Sedaghat and Karimi [24]	10.1016/j.jocn.2020.04.062	Journal of Clinical Neuroscience
Miller Fisher syndrome and polyneuritis cranialis in COVID-19	Gutiérrez-Ortiz et al. [25]	10.1212/WNL.0000000000009619	Neurology

Data Collection and Analysis

Data was collected in the following categories when available:

1. Study design

2. Study country

3. Patient demographics

4. Clinical signs and symptoms- non-neurological

5. Clinical signs and symptoms- neurological

6. Laboratory and radiological findings

7. Treatment

8. Follow-up

We tabulated the data using Microsoft Excel. Statistical analysis was not required, as this is a traditional review. Referencing was done according to guidelines and with EndNote.

This study did not require ethical approval as data was obtained from already available databases, and patients were not directly involved.

Results/analysis

A total of 20 eligible studies were screened and found eligible for data extraction (see Table 1).

**Figure 1:**
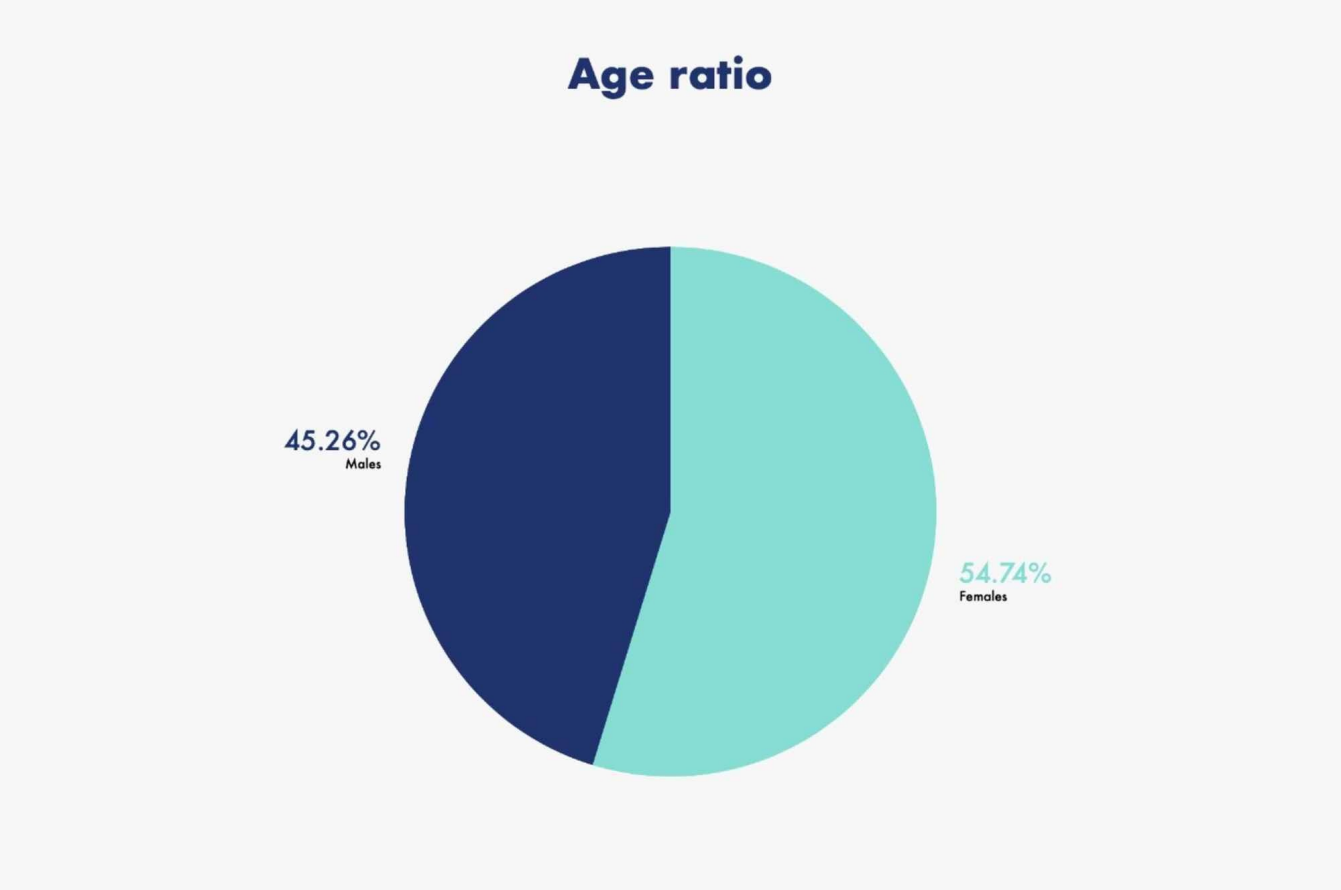
Chart comparing the male and female ratio

The study reports 1,034 COVID-19 positive patients with 468 males (45.26%) and 566 females (54.74%) as seen in Figure 1. The age range of the patients was six weeks to 74 years, with a median age of 44 years - almost all patients presented with neurological and non-neurological symptoms (see Table 2) [6-25].

**Table 2:** Epidemiological characteristics

MEDIAN AGE OF PATIENTS	44 YEARS
NUMBER OF FEMALES	566
NUMBER OF MALES	468
MALE : FEMALE RATIO	0.826 : 1

**Figure 2:**
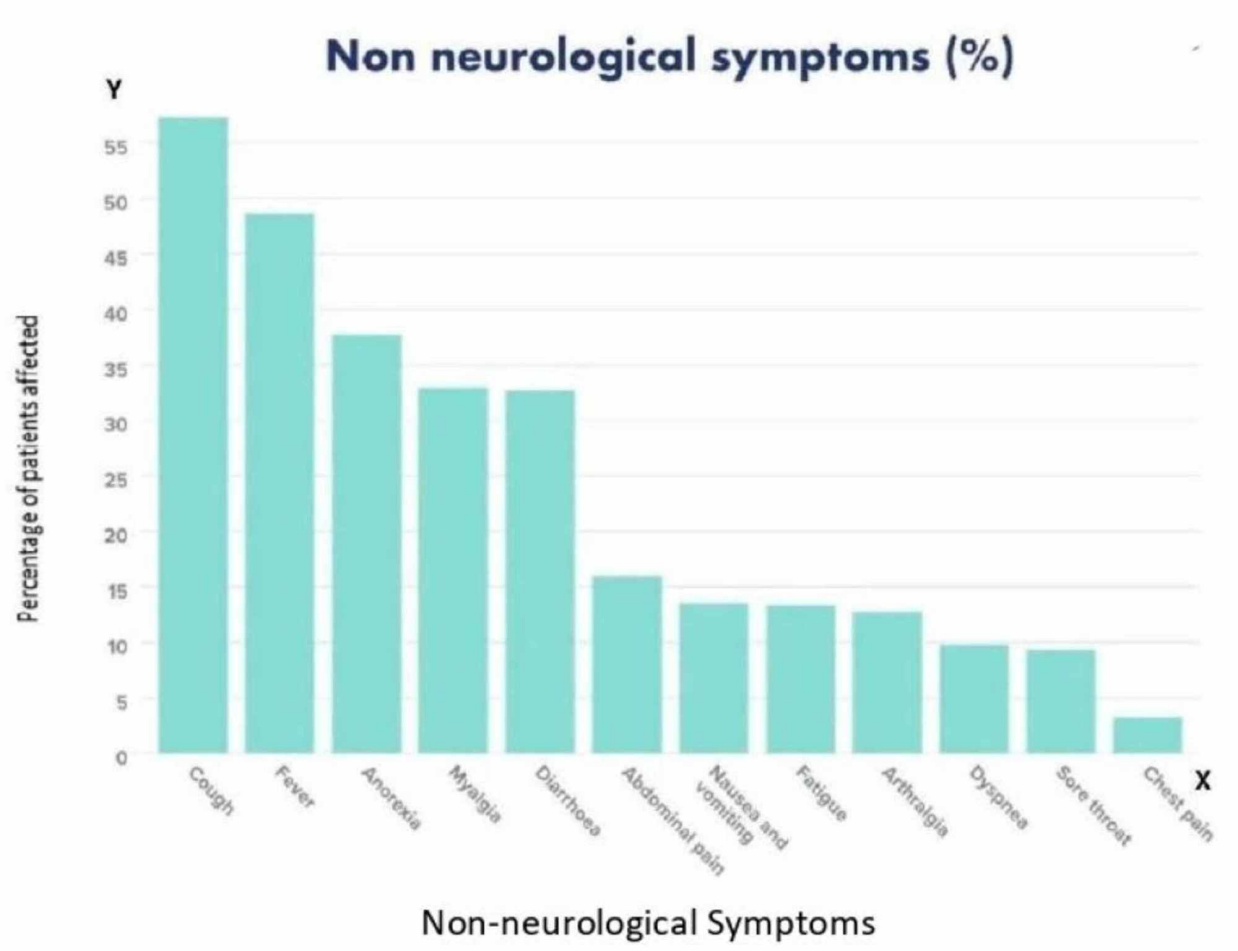
Bar graph showing the various non-neurological symptoms

The most common non-neurological symptoms were fever (48.6%) and coughing (57.37%). Other non-neurological symptoms were diarrhea, anorexia, myalgia, sore throat, dyspnea, chest pain, fatigue, headache, arthralgia, nausea, and vomiting (see Figure 2 and Table 3) [6-25].

**Table 3:** Non-neurological manifestations

NON-NEUROLOGICAL SYMPTOM	NUMBER OF PATIENTS, N = 1034
FEVER	503
COUGH	593
DIARRHEA	338
ANOREXIA	390
MYALGIA	341
SORE THROAT	97
DYSPNEA	101
ARTHRALGIA	132
NAUSEA AND VOMITING	140
CHEST PAIN	34
FATIGUE	139
ABDOMINAL PAIN	165

Among neurological symptoms, headaches were the most common [6-25]. Both central nervous system (CNS) and peripheral nervous system (PNS) symptoms were analyzed, and it was found that a higher number of patients had peripheral symptoms. Headache was seen in 307 patients and dizziness in 64 patients; impaired consciousness and altered mental states were seen in 18 patients [6-25]. Five patients also complained of neuralgia and two patients complained of paresthesia [9,18,19]. Gait abnormality was observed in one patient [19]. Guillain-Barré syndrome (GBS) was the diagnosis in four cases (see Table 4) [13,18,19,24].

**Table 4:** Neurological manifestations

NEUROLOGICAL SYMPTOMS	NUMBER OF PATIENTS, N = 1034
HEADACHE	307
DIZZINESS	64
ALTERED MENTAL STATUS	18
NEURALGIA	5
PARAESTHESIA	2
GAIT ABNORMALITIES	1
OLFACTORY SYMPTOMS	570
AGEUSIA	400
OCULAR SYMPTOMS	12
SENSORINEURAL HEARING LOSS	1

**Figure 3:**
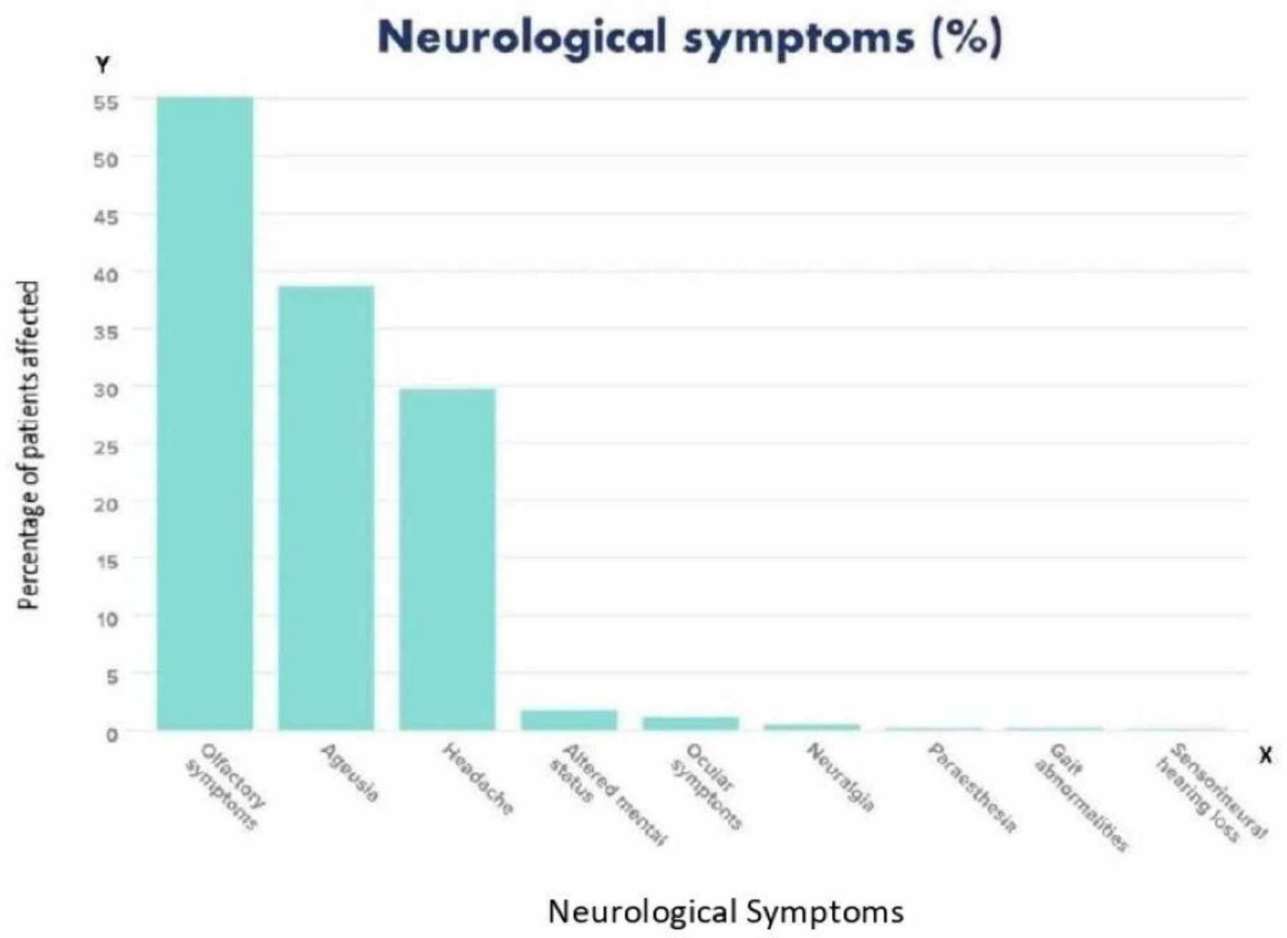
Bar graph showing the various neurological symptoms

Olfactory symptoms were present in 570 patients and mainly included anosmia in 319 patients [6-25]. Hyposomnia was seen in 91 patients. Ageusia was observed in 400 patients and included loss of salty, sweet, sour, and bitter tastes [6-25]. Ocular symptoms were seen in 12 patients and included conjunctivitis, including conjunctival hyperemia in three, chemosis in eight, epiphora in seven, and increased secretions in seven [16]. One patient also presented with sensorineural hearing loss [15]. One patient was found to be encephalopathic, nonverbal, and unable to follow any commands (see Figure 3 and Table 4) [6].

The six-week preterm infant had a witnessed episode of sustained upward gaze associated with bilateral leg stiffening and decreased responsiveness lasting ten seconds with subsequent return to baseline and no hypoxia or vital signs change [20].

Our results show that there is a wide range of symptoms that a COVID-19 patient can present with. Hence, physicians must suspect COVID-19 in those presenting with neurological symptoms as well.

Discussion

Coronaviruses are a group of viruses that can involve and affect multiple organ systems. They can affect both humans and animals [26]. The novel coronavirus SARS-CoV-2 is the causative agent of COVID-19. Coronaviruses are a group of enveloped viruses [26]. The alphacoronavirus and betacoronavirus infect mammals [26,27]. Coronaviruses are spread mainly by the aerosol route. They can also be spread by feco-oral transmission and through fomites. Human coronaviruses primarily target the epithelial cells of the respiratory epithelium, whereas the animal coronaviruses have their main affinity towards the epithelial cells of the digestive tract [28].

Three human coronaviruses produce symptoms that are severe:

1. Middle East respiratory syndrome-related coronavirus (MERS-CoV)

2. Severe acute respiratory syndrome coronavirus (SARS-CoV)

3. Severe acute respiratory syndrome coronavirus 2 (SARS-CoV-2)

All the above three viruses belong to betacoronavirus [27].

Coronaviruses are primarily attracted to the respiratory epithelium [3]. Some studies provide evidence of neuronal involvement of SARS-CoV-2. The nervous tissue expresses the ACE-2 (angiotensin-converting enzyme-2) receptor, which has been detected over the glial and the neural tissues. SARS-CoV-2 has an affinity for the ACE-2 receptors, thus making the nervous system a probable target of SARS-CoV-2 [3].

Route of the Spread of SARS-CoV-2 into the Nervous System

SARS-CoV-2 being a new emergent virus, little is known about its mechanism of action. Because of its genomic similarity with severe acute respiratory syndrome (SARS-CoV) and Middle East respiratory syndrome (MERS-CoV), it is considered to mimic them [29]. In the Mediterranean SARS-CoV infection, the autopsy findings showed evidence of neural tissue involvement [30]. The spread of SARS-CoV-2 through the cribriform plate, which is in close proximity to the olfactory region, has been demonstrated in patients affected with SARS-CoV-2. SARS-CoV-2 can spread through two different pathways [30]. The virus may enter the systemic circulation and enter the cerebral arteries through which it gains access to the brain. The endothelium of the capillaries also contains the ACE-2 receptors [30]. Thus, the virus may get attracted to the ACE-2 receptors and destroy the capillary endothelium, thus breaking the blood-brain barrier and entering the brain. The virus starts multiplying in the brain and can spread along the neural tissue due to the presence of ACE-2 receptors and can cause neural damage without significant inflammation [30]. The accessory pathway through which the SARS-CoV-2 can gain entry to the CNS is through the cribriform plate as it is in close proximity to the olfactory bulb [25].

Manifestation of SARS-CoV-2

After a comprehensive screening of the available literature, a total of 20 eligible studies, which included mostly case series and case reports, were selected, and the patient data was assessed. The study reports a total of 1034 COVID-positive patients, of which 468 were males (45.26%) and 566 were females (54.74%) as seen in Figure 1. The age range of the patients was six weeks to 74 years, with an average age of 44 years as seen in Table 2. The majority of patients in our study (734) were European in origin [7,10,17]. A wide variety of comorbidities such as asthma, hypertension, hypothyroidism, and diabetes mellitus were present. Ground glass opacities were the most common radiological findings. The patients presented with a wide range of neurological and non-neurological symptoms, as summarized below.

All patients were screened for COVID-19 by oropharyngeal swab testing.

Table 3 outlines the non-neurological symptoms of 1034 patients. The most common non-neurological symptoms include fever (48.6%), cough (57.37%), anorexia (37.71%), myalgia (32.97%), and diarrhea (32.68%). Other non-neurological symptoms were throat soreness, dyspnea, chest pain, fatigue, headache, arthralgia, nausea, and vomiting. Chest pains, throat soreness, and dyspnea were seen in very few patients.

Neurological Manifestations of SARS-CoV-2

Table 4 outlines the neurological manifestations of the patients in the study.

Both CNS and PNS symptoms were analyzed, and it was found that more patients had peripheral symptoms. The presentation of olfactory symptoms in SARS-CoV-2-affected patients is due to the fact that the illness spreads through the cribriform plate, which is in close proximity to the olfactory region [30].

GBS was diagnosed in four cases, but the outcomes were not specified [13,18,19,24]. One patient also presented with neurosensory hearing loss [15]. This patient was cared for with standard respiratory care and recovery. There was no observation of a change of hearing loss in this case. Auditory complication due to coronavirus is little mentioned in the literature [15]. A 75-year-old male patient was found to be encephalopathic, nonverbal, and unable to follow any commands [6]. Considering the possibility of subclinical seizures due to an area of encephalomalacia and epileptiform discharges in the right temporal region, anti-epileptic medications were given prophylactically. The patient was treated empirically with vancomycin, meropenem, and acyclovir [6]. A lumbar puncture did not indicate any evidence of central nervous system infection. Due to his progression in symptomatology, he was then tested for COVID-19 and found to be positive [6]. The patient developed respiratory failure, required intubation, and was transferred to the ICU. The patient was started on hydroxychloroquine and lopinavir/ritonavir and was continued on broad-spectrum antibiotics. The patient is critically ill with poor prognosis and currently remains in the ICU [6].

The six-week preterm infant had a witnessed episode of sustained upward gaze associated with bilateral leg stiffening and decreased responsiveness lasting ten seconds, with a subsequent return to baseline, and no hypoxia or vital signs change [20].

A 64-year-old male patient from France with no significant comorbidities who was infected with SARS-CoV-2 presented with paresthesia in feet and hands, had a fever and cough for two days, and developed severe flaccid tetraparesis within three days [18]. Electrodiagnostic tests five days after neurological symptom onset showed a demyelinating pattern in accordance with GBS criteria. On needle examination, no rest-activity was observed [18]. During muscle contraction, only one single motor unit was recorded with a firing rate up to 25 Hz in the right tibialis anterior, the right vastus lateralis, the left first interosseous, and the left deltoideus muscles. On cerebrospinal fluid (CSF) analysis, the protein level was 1.66 g per liter, and the cell count was normal [18]. Anti-gangliosides antibodies were absent in the serum. Biological tests were not in favor of a recent infection with Campylobacter jejuni, Mycoplasma pneumoniae, Salmonella enterica, cytomegalovirus (CMV), Epstein-Barr virus (EBV), herpes simplex virus (HSV) 1 & 2, varicella zoster virus (VZV), Influenza virus A & B, HIV, and hepatitis E [18]. Thoracic CT scan showed only 10%-25% of ground-glass opacities. This was the first case of GBS arising as a complication of COVID-19 infection [18].

## Conclusions

Our results show that there is a wide range of symptoms that can be presented by COVID-19 patients. Hence, physicians must suspect COVID-19 in those presenting with neurological symptoms as well. This systematic review of the current literature on COVID-19 provides insight into some of the atypical manifestations of the disease. With the pandemic continuing to unfold, research is the need of the hour. Even with massive numbers of publications, gaps remain in the understanding of the natural history of the disease. Further studies need to be undertaken in this regard.

## References

[1] Yan CH, Faraji F, Prajapati DP, Boone CE, DeConde AS (2020). Association of chemosensory dysfunction and Covid-19 in patients presenting with influenza-like symptoms. Int Forum Allergy Rhinol.

[2] Hopkins C, Surda P, Kumar N (2020). Presentation of new onset anosmia during the COVID-19 pandemic. Rhinology.

[3] Baig AM, Khaleeq A, Ali U, Syeda H (2020). Evidence of the COVID-19 virus targeting the CNS: tissue distribution, host-virus interaction, and proposed neurotropic mechanisms. ACS Chem Neurosci.

[4] Baig AM (2020). Neurological manifestations in COVID-19 caused by SARS-CoV-2. CNS Neurosci Ther.

[5] Kim JM, Chung YS, Jo HJ (2020). Identification of coronavirus isolated from a patient in Korea with COVID-19. Osong Public Health Res Perspect.

[6] Filatov A, Sharma P, Hindi F, Espinosa PS (2020). Neurological complications of coronavirus disease (COVID- 19): encephalopathy. Cureus.

[7] Lechien JR, Chiesa-Estomba CM, De Siati DR (2020). Olfactory and gustatory dysfunctions as a clinical presentation of mild-to-moderate forms of the coronavirus disease (COVID- 19): a multicenter European study. Eur Arch Otorhinolaryngol.

[8] Gane SB, Kelly C, Hopkins C (2020). Isolated sudden onset anosmia in COVID-19 infection. A novel syndrome?. Rhinology.

[9] Mao L, Wang M, Chen S (2020). Neurological manifestations of hospitalized patients with COVID-19 in Wuhan, China: a retrospective case series study. medRxiv.

[10] Giacomelli A, Pezzati L, Conti F (2020). Self-reported olfactory and taste disorders in patients with severe acute respiratory coronavirus 2 infection: a cross-sectional study. Clin Infect Dis.

[11] Moriguchi T, Harii N, Goto J (2020). A first case of meningitis/encephalitis associated with SARS-Coronavirus-2. Int J Infect Dis.

[12] Poyiadji N, Shahin G, Noujaim D, Stone M, Patel S, Griffith B (2020). COVID-19-associated acute hemorrhagic necrotizing encephalopathy: CT and MRI features. Radiology.

[13] Zhao H, Shen D, Zhou H, Liu J, Chen S (2020). Guillain-Barre syndrome associated with SARS-CoV-2 infection: causality or coincidence?. Lancet Neurol.

[14] Eliezer M, Hautefort C, Hamel A-L, Verillaud B, Herman P, Houdart E, Eloit C (2020). Sudden and complete olfactory loss function as a possible symptom of COVID-19. JAMA Otolaryngol Head Neck Surg.

[15] Sriwijitalai W, Wiwanitkit V (2020). Hearing loss and COVID- 19: a note. Am J Otolaryngol.

[16] Wu P, Duan F, Luo C, Liu Q, Qu X, Liang L, Wu K (2020). Characteristics of ocular findings of patients with coronavirus disease 2019 (COVID-19) in Hubei Province, China. JAMA Ophthalmol.

[17] Spinato G, Fabbris C, Polesel J, Cazzador D, Borsetto D, Hopkins C, Boscolo-Rizzo P (2020). Alterations in smell or taste in mildly symptomatic outpatients with SARS-CoV-2 infection. JAMA.

[18] Camdessanche JP, Morel J, Pozzetto B, Paul S, Tholance Y, Botelho-Nevers E (2020). COVID-19 may induce Guillain-Barre syndrome. Rev Neurol (Paris).

[19] Padroni M, Mastrangelo V, Asioli GM (2020). Guillain-Barré syndrome following COVID- 19: new infection, old complication?. J Neurol.

[20] Dugue R, Cay-Martínez KC, Thakur K (2020). Neurologic manifestations in an infant with COVID-19. Neurology.

[21] Duong L, Xu P, Liu A (2020). Meningoencephalitis without respiratory failure in a young female patient with COVID-19 infection in Downtown Los Angeles, early April. Brain Behav Immun.

[22] Yin R, Feng W, Wang T (2020). Concomitant neurological symptoms observed in a patient diagnosed with coronavirus disease 2019. J Med Virol.

[23] Beltrán-Corbellini Á, Chico-García JL, Martínez-Poles J (2020). Acute-onset smell and taste disorders in the context of Covid-19: a pilot multicenter PCR-based case-control study. Eur J Neurol.

[24] Sedaghat Z, Karimi N (2020). Guillain Barre syndrome associated with COVID-19 infection: a case report. J Clin Neurosci.

[25] Gutiérrez-Ortiz C, Méndez A, Rodrigo-Rey S (2020). Miller Fisher syndrome and polyneuritis cranialis in COVID-19. Neurology.

[26] Decaro N (2011). Alphacoronavirus. The Springer Index of Viruses.

[27] Decaro N (2011). Betacoronavirus. The Springer Index of Viruses.

[28] King AMQ, Adams MJ, Carstens EB, Lefkowitz EJ (2012). Family - Coronaviridae. Virus Taxonomy.

[29] Li YC, Bai WZ, Hashikawa T (2020). The neuroinvasive potential of SARS-CoV2 may play a role in the respiratory failure of COVID-19 patients. J Med Virol.

[30] Toljan K (2020). Letter to the editor regarding the viewpoint “Evidence of the COVID-19 virus targeting the CNS: tissue distribution, host-virus interaction, and proposed neurotropic mechanism”. ACS Chem Neurosci.

